# Classification of Parotid Tumors with Robust Radiomic Features from DCE- and DW-MRI

**DOI:** 10.3390/jimaging11040122

**Published:** 2025-04-17

**Authors:** Francesca Angelone, Silvia Tortora, Francesca Patella, Maria Chiara Bonanno, Maria Teresa Contaldo, Mario Sansone, Gianpaolo Carrafiello, Francesco Amato, Alfonso Maria Ponsiglione

**Affiliations:** 1 Department of Electrical Engineering and Information Technology, University of Naples Federico II, 80125 Naples, Italy; msansone@unina.it (M.S.); framato@unina.it (F.A.); alfonsomaria.ponsiglione@unina.it (A.M.P.); 2 Department of Radiology, San Paolo University Hospital, ASST Santi Paolo e Carlo, 20142 Milan, Italy; silvia.tortora@unimi.it (S.T.); francesca.patella@asst-santipaolocarlo.it (F.P.); mariachiara.bonanno@aphp.fr (M.C.B.); 3 Postgraduation School of Radiodiagnostics, University of Milan, 20122 Milan, Italy; maria.contaldo@unimi.it (M.T.C.); gianpaolo.carrafiello@unimi.it (G.C.); 4 Interventional Radiology Unit, Department of Radiology, Foundation IRCCS Ca’ Granda-Ospedale Maggiore Policlinico, 20122 Milan, Italy; 5 Department of Health Science, Università degli Studi di Milano, 20122 Milan, Italy

**Keywords:** machine learning, parotid tumors, radiomics, robustness

## Abstract

This study aims to evaluate the role of MRI-based radiomic analysis and machine learning using both DWI with multiple B-values and dynamic contrast-enhanced T1-weighted sequences to differentiate benign (B) and malignant (M) parotid tumors. Patients underwent DCE- and DW-MRI. An expert radiologist performed the manual selection of 3D ROIs. Classification of malignant vs. benign parotid tumors was based on radiomic features extracted from DCE-based and DW-based parametric maps. Care was taken in robustness evaluation and the no-bias selection of features. Several classifiers were employed. Sensitivity and specificity ranged from 0.6 to 0.8. The combination of LASSO + neural networks achieved the highest performance (0.76 sensitivity and 0.75 specificity). Our study identified a few robust DCE-based radiomic features with respect to ROI selection that can effectively be adopted in classifying malignant vs. benign parotid tumors.

## 1. Introduction

Salivary gland tumors are relatively rare, constituting 3–11% of all head and neck neoplasms and about 0.2% of all malignancies [1,2]. The global annual incidence of salivary gland tumors is 0.4–13.5 cases per 100,000 individuals [3]. Between 64 and 80% of all salivary gland tumors occur in the parotid gland, with peak incidence in the sixth and seventh decades of life and a male–female ratio of 1.3:1 [1,3,4]. Salivary gland tumors include a wide range of histological subtypes, the majority of which are benign (approximately 80%) [5]. The most common benign tumors are pleomorphic adenoma (A) and Warthin’s tumor (W), while mucoepidermoid carcinoma (M) represents the most frequent malignant subtype [3,4,6].

Accurate preoperative differentiation plays a crucial role in surgical planning. Total parotidectomy may be required for malignant tumors, depending on their location and the histological grade. Pleomorphic adenomas typically require extracapsular dissection [7] due to their high recurrence rate (45–50%) [8,9], while Warthin’s tumors generally warrant simple enucleation or clinical observation [10].

For diagnostic purposes, magnetic resonance imaging (MRI) and fine-needle aspiration (FNA) are essential tools [11,12]. However, FNA has several limitations, including a high rate of non-diagnostic results, sampling errors—such as failure to target the appropriate tissue—and a potential risk of facial nerve palsy [6,13]. MRI should be performed prior to FNA due to the risk of procedure-related bleeding, and the imaging protocol should include a T1-weighted sequence to assess any spontaneous high signal intensity related to intralesional hematic–protein components; a T2-weighted sequence (with and without fat saturation) to compare the signal characteristics of the lesion with the normal parotid gland; diffusion-weighted imaging (DWI) to evaluate the Brownian motion of water molecules; apparent diffusion coefficient (ADC) values derived from two-dimensional (2D) regions of interest (ROIs); and a dynamic contrast-enhanced (DCE) T1-weighted sequence to quantify gadolinium-enhanced signal intensity [6,14]. Despite the complementary roles of MRI and FNA, there may be overlapping features in both cytological findings and imaging characteristics across different histological subtypes [13].

Radiomics is a rapidly emerging field that enables the quantification of phenotypic characteristics from medical images (CT, MRI, US, etc.) using dedicated automated algorithms.

Through imaging, tissues can be characterized non-invasively, and, in some cases, even profound phenotypic differences can be visualized. The result of this analysis consists of quantitative parametric variables which may correlate with tumor histological subtype and have the potential to improve diagnostic accuracy as well as the prediction of the pathological characteristics and the treatment response [6,14,15,16,17,18].

Due to the vast number of available radiomic features, the Imaging Biomarker Standardization Initiative (IBSI) [19] has reviewed the main feature definitions and developed an open-source Python (version 3.6) package [20] to enhance study reproducibility.

Given the high number of features that can be extracted from each parametric map in DCE and DW-MRI examinations, the total number of computed variables can easily reach the thousands. This introduces the risk of feature selection bias [21], a phenomenon also observed in genomic studies.

Moreover, feature reproducibility (robustness) remains a key issue for clinical applicability. In particular, it has been shown that radiomic features can be highly sensitive to ROI definition and placement [22,23].

Radiomics is based on the concept that distinct imaging features among different disease phenotypes may help predict diagnosis and prognosis, including treatment response. If radiomics could accurately differentiate between benign (B) or malignant (M) salivary gland tumors, it would offer valuable support for reducing the need for invasive diagnostic procedures (such as FNA or biopsy) and for tailoring therapies accordingly [24].

Machine learning, through the processing of large-scale medical data, is increasingly promoting the development of personalized medicine across a variety of clinical domains, particularly in predicting tumor diagnosis and prognosis [25,26,27]. It has already become a fundamental tool in medical data analysis, enabling computer-aided solutions to support clinical decision-making.

Radiomics and artificial intelligence have the potential to significantly improve the preoperative differentiation of parotid tumors compared to conventional methods and could be extended to other clinical applications.

Given the critical importance of accurate preoperative characterization for surgical planning—and the known limitations of FNA currently used in clinical practice for this purpose—the development of reliable, non-invasive, computer-assisted diagnostic systems is essential.

The primary aim of this study was to develop a radiomics-based model capable of classifying parotid gland tumors as benign or malignant, using features extracted from MRI sequences, specifically DCE and DWI. Particular attention was given to ensuring analytical robustness and minimizing potential sources of bias. To account for inter- and intra-operator variability and to evaluate the reproducibility of radiomic features, multiple regions of interest (ROIs) were synthetically generated by applying several transformations to the initial manually segmented ROI. Subsequently, a selection of features was included in a cross-validation process to avoid selection bias.

This analysis could offer useful insights into the clinical interpretability of radiomic features in relation to tumor classification.

## 2. Materials and Methods

### 2.1. Patient Population

This was a retrospective study involving 41 patients (19 male, 22 female; age range: 18–88 years; median age: 59.4 years) with parotid gland lesions (10 W, 15 A, and 18 M) who underwent neck MRI at San Paolo Hospital, Milan (Italy), between March 2017 and July 2023. All patients provided written informed consent for MRI examination performed within the diagnostic workup of parotid gland lesions.

The inclusion criterion was defined as the presence of at least one salivary gland lesion detectable on MRI, suitable for segmentation and radiomic analysis.

Exclusion criteria included FNA performed within one month prior to MRI; nodule diameter less than 1 cm (to reduce partial volume effects and segmentation variability, which are more significant in subcentimeter lesions); and MRI examinations lacking either multiple b-values on DWI or DCE sequences.

This study was approved by our internal review board and was conducted in accordance with the ethical statements of the Declaration of Helsinki.

### 2.2. MR Imaging

The MRI was performed using a 1.5 T scanner (OPTIMA 450 W, GE Healthcare, Little Chalfont, UK) equipped with 36 multichannel coils, gradients of 40, 40, and 45 mT/m along the *x*, *y*, and *z* axes, and a slew rate of 200 mT/m/ms. Scanning parameters are detailed in Table 1. Patients underwent DWI with 11 b-values: 0, 10, 20, 30, 50, 80, 100, 200, 300, 400, and 800 s/mm^2^. The in-plane resolution was approximately 0.8 × 0.8 mm, with a slice thickness of 3 mm, no interslice gap, and a matrix size of 256 × 256.

After administration of a paramagnetic contrast agent (Gadobutrol, Gadovist; Bayer Schering Pharma, Leverkusen, Germany) at a dose of 0.1 mg/kg, DCE MRI was performed, consisting of one pre-contrast acquisition followed by 10 post-contrast acquisitions.

The temporal resolution was approximately 38 s per acquisition, resulting in a total acquisition time of about 6.5 min. The in-plane resolution was approximately 0.5 × 0.5 mm, with a slice thickness of ~1 mm, no interslice gap, and a matrix size of 512 × 512.

### 2.3. Segmentation

**Figure 1:**
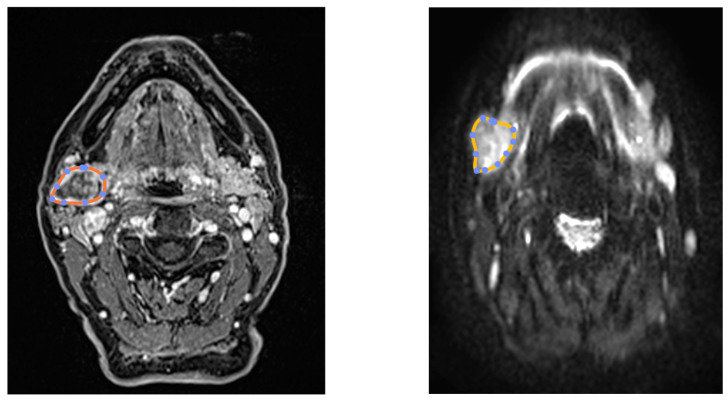
Segmentation of dynamic DCE and DWI.

Three-dimensional segmentation of the target nodules was performed using Horos (available at https://horosproject.org, accessed on 2 February 2025) [28] by two radiologists (MB and FP), on both DWI and DCE-MRI sequences (Figure 1). The entire dynamic sequence was utilized for segmentation, as patient motion was minimal and deemed negligible.

Segmentations were exported from Horos in XML format, subsequently imported into Python [29] and converted to NIfTI format [30] for further processing.

### 2.4. Artificial ROI Generation for Robustness Evaluation

To evaluate the robustness of radiomic features (see Section 2.8) with respect to ROI selection, accounting for both inter- and intra-observer segmentation variability, several artificial ROIs were artificially generated starting from the original manually defined ROI. Specifically, fifteen artificial ROIs were generated using the following manipulations:

(1) Axial rotation of 10°, 20°, and 30° degrees clockwise and counterclockwise around the barycenter of the original ROI (six ROIs);
(2) Dilations using a 3D structured element of connectivity of 1 or 2 pixels (two ROIs);
(3) Erosion using a structured element of 1 pixel (one ROI);
(4) Translation of 1 pixel in 3 orthogonal directions, both forward and backward (six ROIs).

Artificial ROI generation was implemented using the scipy.ndimage module from the SciPy library in Python (https://scipy.org/, accessed on 2 February 2025).

### 2.5. Parametric Maps

**Figure 2:**
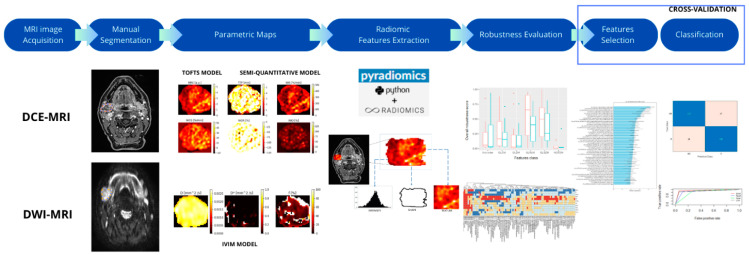
Block diagram of the pipeline described in the text. After manual ROI selection, parametric maps were computed, voxel-by-voxel, from DCE-MR and DW-MR data (Section 2.6). From these maps, radiomic features were extracted (Section 2.7). Radiomic feature robustness with respect to ROI selection was evaluated by applying several operations on manual ROI (morphological, translations, rotations, etc.) (Section 2.5 and Section 2.8). Robust features were used in the subsequent selection–classification phase: this phase was cross-validated to avoid feature selection bias (Section 2.5 and Section 2.9).

**Figure 3:**
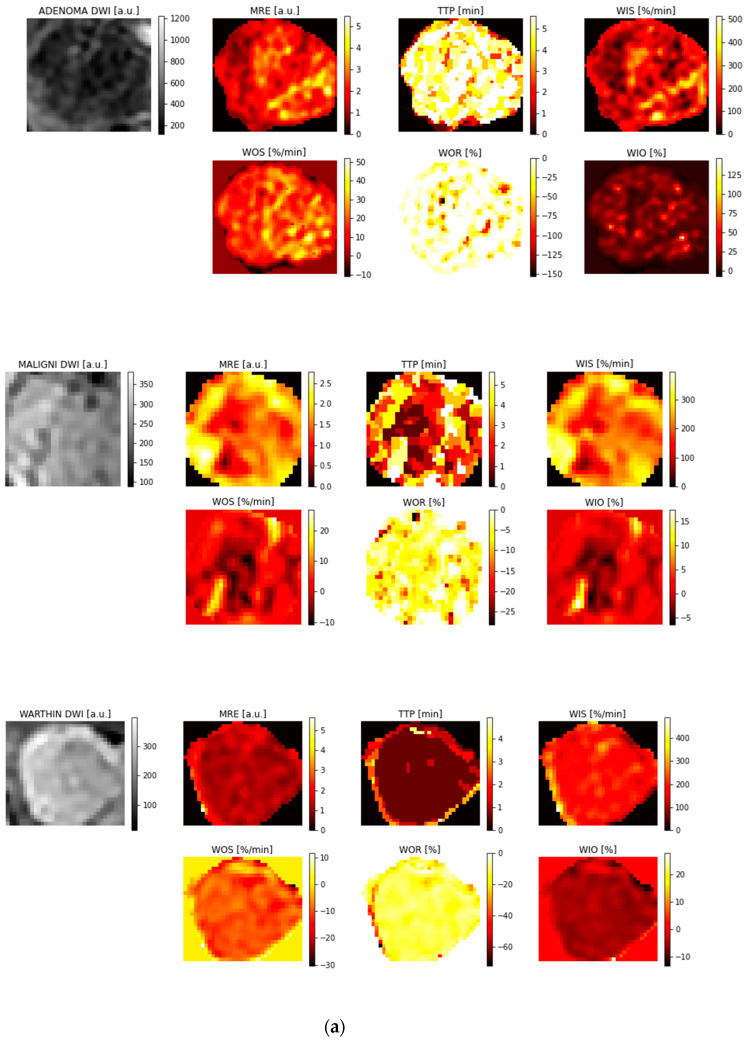

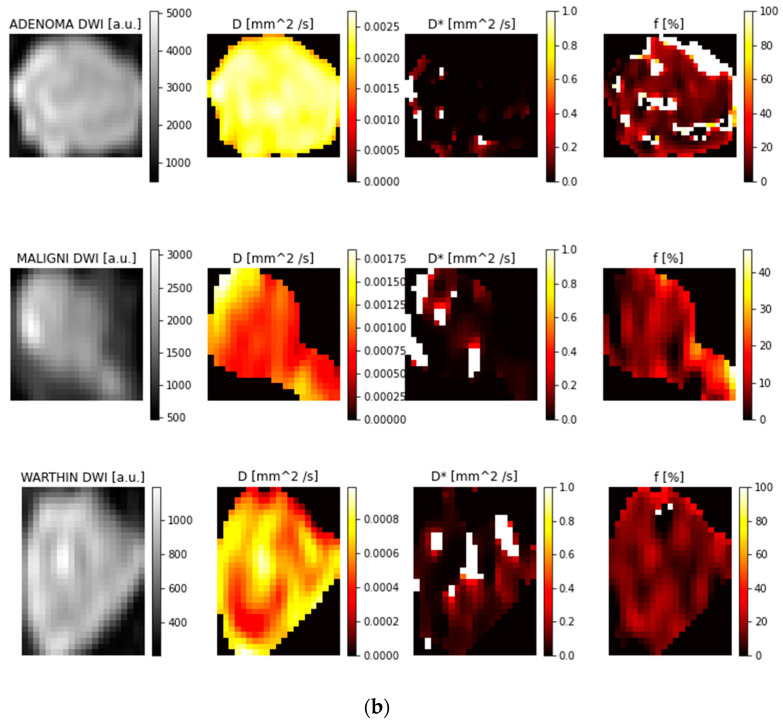
(**a**) Intermediate results. Examples of semi-quantitative parametric maps computed from ROIs of patients representative of each class. (**b**) Example of IVIM parametric maps computed from ROIs of patients representative of each class.

In this study, we applied the processing pipeline described in Figure 2. For each 3D ROI (either the original manually segmented one or the artificially generated variants), parametric maps were computed on a voxel-by-voxel basis. For dynamic contrast-enhanced MRI (DCE-MRI), six model-free parametric maps (model-free parameters) were computed using in-house Python code: maximum relative enhancement (MRE), time to peak (TTP), wash-in slope (WIS), wash-out slope (WOS), wash-out ratio (WOR), and wash-in–out ratio (WIO)—defined as the ratio between wash-in and wash-out slopes. Moreover, three parametric maps were derived from Tofts’ model [4] (model-based parameters): capillary permeability (perfusion coefficient, Ktrans), extracellular volume fraction (Ve), and plasma volume fraction (Vp). Thus, a total of nine DCE maps were available. In addition, for DWI, the model-based parametric maps were computed based on intravoxel incoherent motion (IVIM) modeling [5,6]: the true diffusion coefficient (D), the pseudo-diffusion coefficient (D*), and the perfusion fraction (f), resulting in three DWI parametric maps in total. Examples of calculated parametric maps are reported in Figure 3.

Further methodological details on Tofts’ model, IVIM modeling, and semi-quantitative parameters can be found in the work by Patella F. et al. [13].

### 2.6. Radiomic Features

For each parametric map, 93 radiomic features were computed using the IBSI-compliant software package *PyRadiomics* (version 3.1.0) [19,20] implemented in Python. The feature classes included first-order statistics (first order, 18 features), Gray Level Co-occurrence Matrix (GLCM, 24), Gray Level Run Length Matrix (GLRLM, 16), Gray Level Size Zone Matrix (GLSZM, 16), Neighbouring Gray Tone Difference Matrix (NGTDM, 5), and Gray Level Dependence Matrix (GLDM, 14). In total, 93 features were computed for each of the parametric maps, resulting in 1116 radiomic features per patient.

To reference individual features, the following naming convention was adopted in this manuscript: [feature-group]_[feature-name].[map-name], for example, *glcm_Idmn.VP*, *firstorder_Entropy.MRE.*

### 2.7. Evaluation of Feature Reproducibility

**Figure 4:**
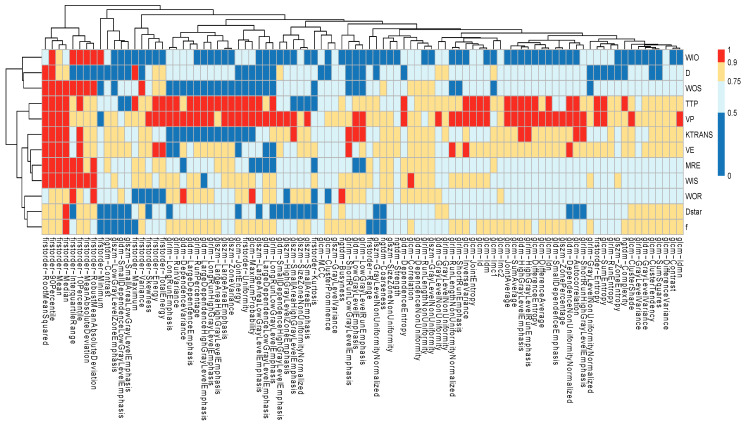
Heatmap of intra-class correlation coefficient (ICC) for radiomic feature maps. ICC values from 0 to 0.5 are commonly considered ‘poor’ agreement; values between 0.5 and 0.7 are considered ‘moderate’ agreement; values between 0.75 and 0.9 are considered ‘good’ agreement; values between 0.9 and 1 are considered excellent agreement.

The intra-class correlation coefficient [31] (ICC) was calculated to assess the reproducibility of radiomic features in relation to ROI selection. Reproducibility was defined as the degree of ‘agreement’ among feature values extracted from the different ROIs [22,23,31]. ICC values were interpreted according to commonly accepted thresholds: values between 0 and 0.5 indicated “*poor*” agreement, 0.5 to 0.75 “*moderate*” agreement, 0.75 to 0.9 “*good*” agreement, and 0.9 to 1 “*excellent*” agreement. The results of the reproducibility analysis are reported in Figure 4. Only features demonstrating ‘excellent’ agreement (ICC > 0.9) were retained for further analysis.

### 2.8. Feature Selection

To minimize selection bias [21], the radiomic feature selection process was integrated into the cross-validation framework (Figure 2). For each classifier (Figure 2), features deemed most relevant, i.e., those most strongly associated with tumor classes, were selected from the pool of robust features identified in the previous phase (Section 2.8). The strength of association between radiomic features and tumor types (pleomorphic adenoma, Warthin’s tumor, and malignant tumors) was assessed using four commonly adopted feature selection methods: AUC-ROC analysis, ReliefF, LASSO, and backward selection. For a general overview of these algorithms, refer to [32].

In AUC-ROC analysis, the three-class classification problem was decomposed into multiple binary subproblems, and the average ROC across all subproblems was computed for each feature. Features with an average ROC greater than 0.8 were considered strongly associated with tumor class.

The ReliefF algorithm provides a ranking of the features based on their ability to discriminate between tumor classes. To obtain a robust estimate of the null distribution, class labels were randomly shuffled 500 times. Features with a ReliefF ranking above the 95th percentile were considered strongly associated with tumor class [33].

LASSO is a type of linear modeling with a penalized approach that produces sparse, non-zero coefficients for the radiomic features [34]. Backward selection starts with the whole set of features and iteratively removes one feature at a time based on a suitable classification accuracy metric [33]. It should be noticed that AUC-ROC analysis and ReliefF algorithms perform univariate filtering, evaluating each feature independently, whereas LASSO and backward elimination consider potential interactions among features.

The entire feature selection procedure was included within the cross-validation process to avoid feature selection bias. Specifically, the training set was divided into 5 equally sized folds; feature selection and model training were performed on 4 folds while the remaining fold was used for performance valuation (Figure 2 and Section 2.5).

### 2.9. Classification and Accuracy Evaluation

Classifier training and feature selection were performed in R [35] using the CARET package [29,36,37]. Four widely used classification algorithms were applied (Figure 2): Linear Discriminant Analysis (LDA), k-nearest-neighbors (k-NN), Support Vector Machines (SVMs), and Neural Networks (NNETs). For additional details on these classifiers, refer to [15].

Parameter tuning for each classifier (number of neighbors for kNN, complexity parameter for SVMs, number of hidden neurons, and decay constant for NNETs) was performed by searching over a predefined grid of candidate values. Performance was evaluated using repeated 5-fold cross-validation with 100 repetitions. To ensure a fair comparison, all classifiers were trained and tested on the same sets of folds, randomly defined in advance.

Classification performance was evaluated using accuracy, sensitivity, and specificity with respect to the detection of malignant tumors (binary classification: malignant vs. non-malignant cases).

## 3. Results

### 3.1. ROI Selection and Parametric Maps

Figure 3a,b show examples of parametric maps computed on ROIs from patients representative of each tumor class.

### 3.2. Feature Reproducibility

Feature selection and classification were performed exclusively on features that were reproducible with respect to ROI selection. Accordingly, only radiomic features showing ‘excellent’ agreement (ICC > 0.9) were included in the classification analysis, constituting the robust feature set. As shown in Figure 4, this corresponds to the 155 features highlighted with red cells.

### 3.3. Feature Selection Results

**Figure 5:**
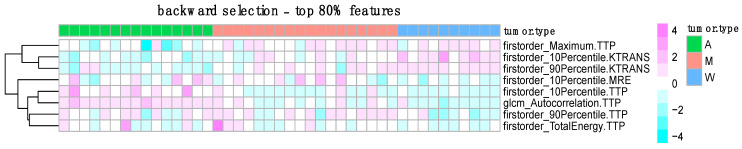
Heatmap representation of radiomic feature values and tumor type. Each column in the matrix represents a patient and each row a feature. Cell colors represent (normalized) radiomic values. Rows are grouped. Adenoma patients have negative values of a group of three features (firstorder_Maximum.TTP, firstorder_10Percentile.KTRANS, firstorder_90Percentile.KTRANS), while the same features have higher values in the Warthin group; malignant patients do not show a clear pattern.

In Figure 5, maps of radiomic features, selected by backward selection in at least 80% of cross-validation iterations, are shown for each patient. This figure highlights a potential association between a small subset of features and tumor class. First-order features from MRE, TTP, and KTRANS maps, along with second-order features from TTP, appear to be associated with pleomorphic adenoma and Warthin’s tumor. However, the association with malignant tumors is less evident.

### 3.4. Classification Performance

**Figure 6:**
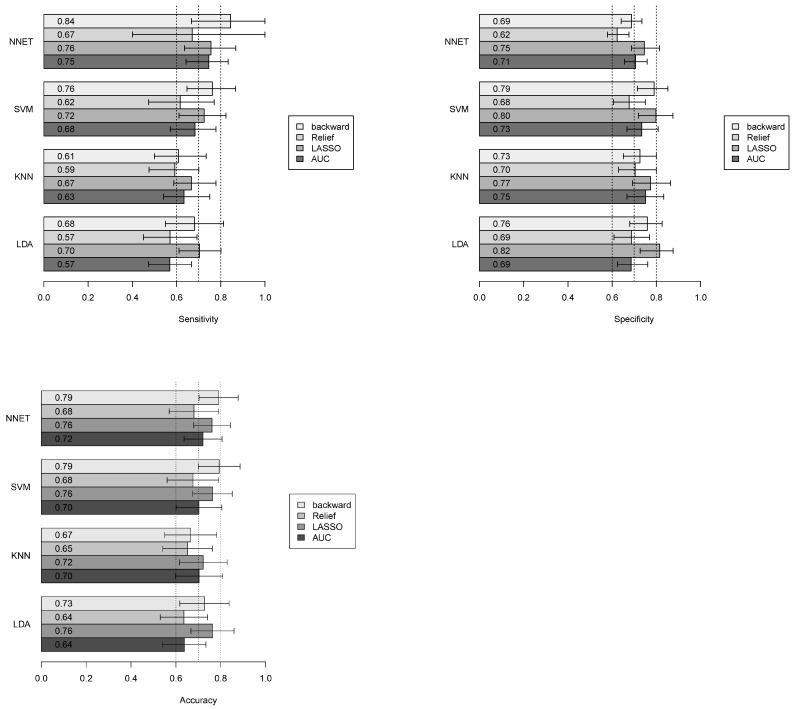
Accuracy, sensitivity, and specificity of different classifiers combined with different feature selection methods. Results are presented as average values across 100 repetitions of 5-fold cross-validation, with 5th–95th percentile intervals as approximate confidence ranges. Vertical dotted lines at 0.6, 0.7, and 0.8 serve as visual performance benchmarks.

Malignant vs. benign classification was performed on an approximately balanced dataset (18 malignant, 25 benign). Figure 6 reports the accuracy, sensitivity, and specificity of the different classifiers combined with various feature selection methods. For each classifier, average performance values across 100 repetitions of 5-fold cross-validation are shown. To illustrate variability, 5th to 95th percentile intervals are also reported. As a visual reference, vertical dotted lines indicating thresholds of 0.6, 0.7, and 0.8 for sensitivity, accuracy, and specificity are superimposed.

## 4. Discussion

In this study, we assessed the classification performance—specifically accuracy, sensitivity, and specificity—of radiomic features extracted from MRI parametric maps (DCE and DWI) for differentiating malignant from benign parotid tumors.

Despite the relatively small sample size, which reflects the rarity of this pathology, particular attention was dedicated to identifying robust and reproducible radiomic features (Figure 4). To minimize feature selection bias, the entire pipeline—from feature selection to classification—was performed within a cross-validation framework (Figure 2). Classifier performance was further evaluated through repeated cross-validation, ensuring consistency and reliability of the results (Figure 6).

Our results indicate that all the classifiers evaluated achieved comparable performance, with accuracy, sensitivity, and specificity generally ranging between 0.6 and 0.8. A greater variability was observed in sensitivity compared to specificity. The best-performing model across all three metrics was the Neural Networks combined with LASSO feature selection (accuracy: 0.76; sensitivity: 0.76; specificity: 0.75). As shown in Figure 6, both LASSO and backward selection, when combined with any of the classifiers, often achieved higher performance. This may be due to their ability to retain interactions among features, whereas AUC-ROC and ReliefF perform univariate filtering. No single classifier demonstrated a clear advantage over the others. Although Neural Networks showed the highest sensitivity, this came at the cost of slightly lower specificity. Furthermore, the substantial overlap of confidence intervals indicates a high degree of variability and reinforces the finding that classifier performance is largely comparable.

From Figure 5, an interesting pattern emerges regarding the most frequently selected features using backward selection (selected in ≥80% of cross-validation iterations). These include first-order features from MRE, TTP, and Ktrans maps, as well as second-order features from TTP. Adenomas appear to exhibit higher values for some of these features, while Warthin’s tumors show lower values. Malignant tumors, however, do not display a consistent association with these features. This observation may help explain the modest sensitivity achieved for malignancy: the most reproducible features do not appear to be strongly linked to malignant tumors.

Figure 4 also provides insight into the reproducibility of IVIM-based parametric maps. Only a very limited number of robust features (ICC > 0.9) were identified from D, D*, and f maps. As a result, no IVIM-derived features were included among the final selected features shown in Figure 5. This suggests that, despite the clinical relevance of diffusion-based parameters, further work is needed to improve the reproducibility of ROI segmentation and to enable automated radiomic feature extraction from DW-MR images.

The “robust” radiomics features evaluated in our study are not necessarily robust for a real-world multicenter setting, as we only assessed reproducibility by perturbing the ROIs. Features that are reproducible across different segmentations do not guarantee true robustness, which also requires evaluating the effects of variations in patient positioning, image acquisition protocols, and segmentation methods—all of which can influence each feature to varying degrees [38,39,40].

In agreement with the previous study by Patella et al., adenomas and Warthin’s are well distinguishable using IVIM-based radiomics [13]. Qi et al. built a radiomics nomogram with good performance in differentiating benign from malignant tumors and adenomas from malignant tumors, but relatively lower performance in distinguishing Warthin’s tumors from malignancies [6]. This may be attributed to the intrinsic heterogeneity of Warthin’s tumors, characterized by high cellularity, stromal density, and vascularization with elevated microvessel density, which can mimic low-grade malignant tumors [22]. Similarly, Gabelloni and Piludu, in their radiomics studies based on features extracted from T2-weighted images and ADC maps, also reported relatively low accuracy in differentiating Warthin’s tumors from malignant ones [14,41].

Kazerooni et al. applied radiomics and machine learning in a cohort of 31 patients (8 malignant), extracting features from T2-weighted images, ADC maps, and the late phase of DCE-MRI. Using a Support Vector Machine (SVM), they reported 100% accuracy in classification [42].

Compared to our study, their dataset was smaller and included fewer malignant cases (8 vs. 18), with the same number of benign cases (23 vs. 25), making their reported performance likely less reliable than ours. Furthermore, their radiomic features were extracted using in-house software less standardized than the *PyRadiomics (version 3.1.0)* package adopted in our study, based on the IBSI framework, currently considered the reference standard for radiomic feature extraction [19]. Additionally, their cross-validation method was a “leave-one-out” type, whereas we adopted k-fold cross-validation, which provides a more reliable estimate of classification error.

There is increasing interest in the clinical applicability of radiomics and machine learning, as reflected in the recent literature [5,6,14,43]. However, no consensus has yet been reached regarding the most appropriate MRI sequences for radiomic analysis of salivary gland tumors [43], and the lack of external validation in many studies limits the assessment of model reliability. Moreover, standardized software validated according to IBSI guidelines has only been adopted in a limited number of studies [14].

This study has several limitations. First, it is a single-center retrospective study, which may introduce bias and confounding factors, particularly affecting external validity and increasing the risk of selection bias [44,45].

Second, we included only nodules larger than 1 cm, in order to minimize partial volume effects and segmentation variability, both of which are more pronounced in subcentimeter lesions and may compromise the robustness of radiomic feature extraction. Future research will extend the analysis to smaller nodules by optimizing segmentation strategies and refining feature selection methods, with the aim of improving model reliability in more complex cases.

Third, we limited the analysis to features with excellent reproducibility (ICC > 0.9). While including features with moderate reproducibility might improve classification performance, we believe that having robust features in terms of ROI selection is important for clinical use.

Furthermore, the dataset was relatively small (43 patients), which reflects the rarity of the disease (0.4–13.5 cases per 100,000 individuals). Nevertheless, great care was taken in designing the cross-validation strategy, providing reasonable confidence in the reproducibility of our results. However, the absence of an external, independent test set remains a limitation in the evaluation of model generalizability.

Finally, only a limited number of classifiers and feature selection methods were considered. However, we believe that the selected methods are widely used and offer a reasonable indication of the performance achievable using the pipeline described in Figure 2. Future studies should aim to expand the dataset, explore a broader set of radiomic features, and include preprocessing transformations prior to feature extraction to further enhance model performance [46,47].

## 5. Conclusions

This study highlights the potential of radiomics and machine learning for classifying parotid tumors using MRI-derived radiomic features. By focusing on the reproducible radiomic features from DCE and DW-MRI, specifically with respect to small ROI segmentation, we aimed to reduce feature selection bias and enhance diagnostic accuracy. Our findings demonstrate that multiple classifiers can achieve sensitivity and specificity values ranging from 0.57 to 0.84, with the combination of LASSO and Neural Networks providing the best performance. Although the results are encouraging, further validation on larger multicenter datasets is needed. Future research should aim to refine robust feature selection strategies, incorporate additional imaging modalities, and perform external validations to strengthen the clinical applicability of radiomics-based classification in the diagnosis of parotid tumors.

## Figures and Tables

**Table 1:** MRI sequence parameters. WI: diffusion-weighted image; EPI: ecoplanar impulse; FA: flip angle; GRE: gradient echo; SE: spin echo; ST: slice thickness; TE: echo time; TR: repetition time.

Sequence	Orientation	TR/TE (ms)	FA (deg.)	Size Image (mm × mm)	Acquisition Matrix	ST/Gap (mm/mm)
T2 2D SE	Axial	7963/129	160	512 × 512	512 × 204	3/0.4
T2 2D SE	Coronal	7963/129	160	512 × 512	384 × 384	3/0.4
T1 2D SE	Axial	500/15	160	512 × 512	512 × 204	3/0.4
DWI EPI	Axial	5290/77	90	256 × 256	140 × 70	3/3
T1 3D GRE	Axial	6/3	12	512 × 512	256 × 256	3/0.8

## Data Availability

The datasets used and/or analyzed during the current study are available from the corresponding author on reasonable request.
